# Anti-AAV Antibodies in AAV Gene Therapy: Current Challenges and Possible Solutions

**DOI:** 10.3389/fimmu.2021.658399

**Published:** 2021-03-17

**Authors:** Thomas Weber

**Affiliations:** ^1^Cardiovascular Institute, Icahn School of Medicine at Mount Sinai, New York, NY, United States; ^2^Graduate School of Biomedical Sciences, Icahn School of Medicine at Mount Sinai, New York, NY, United States

**Keywords:** gene therapy, aav, antibodies, antibody assays, removal of antibodies, removal of inhibitors, adeno-associated virus, prevalence of antibodies

## Abstract

Adeno-associated virus (AAV) vector-based gene therapy is currently the only *in vivo* gene therapy approved in the US and Europe. The recent tragic death of three children in a clinical trial to treat X-Linked Myotubular Myopathy by delivering myotubularin with an AAV8 vector notwithstanding, AAV remains a highly promising therapeutic gene delivery platform. But the successful use of AAV vectors to treat an increasing number of diseases also makes establishing protocols to determine therapeutically relevant titers of pre-existing anti-AAV antibodies and approaches to deplete those antibodies more urgent than ever. In this mini review, I will briefly discuss (i) our knowledge regarding the prevalence of anti-AAV antibodies, (ii) the challenges to measure those antibodies by methods that are most predictive of their influence on therapeutic efficacy of AAV gene transfer, and (iii) approaches to overcome the formidable hurdle that anti-AAV antibodies pose to the successful clinical use of AAV gene therapy.

## Introduction

Thirty years ago, the Chicago Tribune published a front-page article entitled “Gene therapy poised to reinvent medicine.” While, after three humbling decades of intense research, we have not achieved this lofty goal, it is safe to say that we made tremendous progress toward establishing gene therapy as an important tool to treat both inherited as well as acquired diseases. Among the currently available gene therapy platforms, vectors based on adeno-associated virus (AAV) have clearly emerged as one of the most promising gene delivery vehicles.

In fact, only three *in vivo* gene therapy treatments have been approved by regulatory agencies for clinical use (1). Notably, all three of these therapies are based on AAV vectors. Whereas the treatment of lipoprotein lipase deficiency with an AAV-based vector to deliver the defective protein was the first to be approved by the European Medicines Agency (EMA) in 2012, it is currently no longer on the market owing to a lack of demand. In contrast, Luxturna (2) to treat the early childhood blindness disease Leber's congenital amaurosis type 2 is in clinical use in both the US and Europe (1), whereas Zolgensma (1) to treat spinal muscular atrophy (SMA)—a horrific disease that causes the death of most children afflicted by it before age 2—has been approved by the US Food and Drug Administration (FDA) in 2019 (1).

The success of AAV as the leading gene delivery modality is based on a multitude of factors: (i) Even wild-type AAV has not been shown to cause any disease, (ii) In contrast to other currently available gene delivery methods, in non-dividing or very slowly dividing cells AAV results in the long-term expression of the therapeutic payload, even though the AAV vector DNA persists mostly as extrachromosomal episomes (3), (iii) AAV vectors are not strongly immunogenic, most likely because the only viral elements in a therapeutic AAV genome are the two inverted terminal repeats. As a result, AAV vectors do not express any viral proteins, which dramatically reduces the presentation of viral peptides on MHCI complexes thus strongly reducing the incidence and intensity of a cellular immune response against transduced cells (4).

Until recently, AAV gene therapy had an unblemished safety profile (5). Sadly, this unparalleled safety record has recently been shattered by the tragic death of three children in a trial (NCT03199469) aimed at treating X-linked myotubular myopathy (XLMTM) with an AAV8 vector promoting the expression of functional MTM1. This trial is currently on hold, and the cause of the deaths of the three children is under active investigation [Wilson and Flotte (6) and references cited therein].

One of the drawbacks of AAV is its limited cargo capacity of ~5 kb. Even though the median size of a human protein is only 375 amino acids (7), the coding sequences of larger proteins naturally harbor more mutations. Consequently, many proteins associated with inherited diseases cannot be expressed with a single AAV vector because the size of an expression cassette for a functional protein exceeds the packaging capacity of AAV.

As mentioned above, wild-type AAV is an apparently non-pathogenic virus. However, infection with wild-type AAV is very common and depending on the serotype (and assay used, vide infra) 30–60% of all individuals harbor antibodies that neutralize AAV transduction (8). Interestingly, however, even when using the same assay, significant differences in the prevalence of neutralizing antibodies (NAbs) exist (9, 10). In fact, Greenberg et al. (9) showed that within the US the prevalence of antibodies against AAV1 varied from 32% in Wisconsin to 67% in South Carolina, and in Europe 48% of people in Sweden harbor NAbs against AAV1 in contrast to 79% NAb positive people in Poland and Hungary. Another challenge for the use of AAV gene therapy in patients with NAbs against a specific AAV serotype is the fact that there is substantial cross-reactivity among the AAV serotypes (8, 10, 11).

## Assays Measuring Neutralizing and Total Anti-AAV Antibody Levels

In the vast majority of past or ongoing AAV gene therapy clinical trials using AAV vectors the presence of NAbs (determined in an *in vitro* assay) is/was one of the exclusion criteria. While this makes inherent sense, the absence or presence of NAbs is ill defined. The *in vitro* assays employ an AAV reporter vector (usually carrying a luciferase expression cassette or GFP) of the serotype used in the respective clinical trial. Pre-treatment patient serum/plasma samples are then serially diluted and incubated with an AAV reporter virus with a pre-determined number of vector genomes. After addition of the virus to cells (most often HEK293 cells) and incubation for a defined amount of time, the expression of the reporter protein is measured for each serum dilution as well as samples that did not receive any patient serum. Expression levels of each serum dilution sample are then normalized to the no-patient serum control. In general, the neutralizing titer is defined as the highest dilution that reduces transduction by ≥50%, although other levels of reduction have been used as well [e.g., 29% (12)]. However, the assay conditions vary widely not only for preclinical work (8) but also for clinical trials. For instance, for their hemophilia A trial (NCT02576795) (13), Biomarin only enrolled patients that had no neutralizing antibodies against AAV5. Their NAb assay used 25,000 vector genome containing particles per cell (14). Uniqure, on the other hand, used a GFP based assay for their hemophilia B trial (NCT02396342) (15) without reporting the MOI. For another hemophilia B trial (NCT03489291) (16) they used a highly sensitive luciferase assay (12) with an MOI of 378.4. So, even if all assay conditions were absolutely identical, the mere use of an MOI 25,000 and 378.4 would yield dramatically different (>66-fold) NAb titers.

Unfortunately, the AAV dose/MOI is not the only parameter that can influence NAb titers. Two other critically important parameters are the purity of the vector preparation, especially as it relates to the absence or presence of monomeric or oligomeric capsid proteins. Moreover, depending on the vector production method and purification scheme, the final vector preparations can also contain empty viral particles, particles with truncated vector genomes or particles with plasmid or genomic DNA. All of those particles are essentially inactive virus, and their presence in an NAb assay will artificially lower the NAb titer (17). It is also noteworthy to point out that so-called “neutralizing antibody assays” also incorporate inhibition by other factors in the serum [e.g., galectin-3 binding protein (18)] that inhibit transduction. Hence, the terms neutralizing or inhibitory factors is more appropriate. Without doubt, the most critical parameters affecting NAb titers is the AAV dose and serum volume. Therefore, I propose that, in the future, the field should report the presence of NAbs not as NAb titers but rather report the number of AAV particles that are neutralized per μl serum (or plasma). Not only would this facilitate the comparison of NAb assay results among different labs, it would also include non-antibody inhibitory factors. Of course, reporting the number of AAV particles that can be neutralized per μl serum (or plasma) doesn't eliminate variations obtained with different neutralizing assays. However, in the absence of a *very* detailed description of the methods and materials used for NAb assays in every publication, this is the most accurate unit to report neutralizing factors in serum (or plasma).

There are several reports that use an *in vivo* assay to determine neutralization by human or non-human primate (NHP) sera [e.g., (19–21)]. These assays, which rely on the passive immunization of mice with human or NHP sera, appear to be much more sensitive than most of the currently used *in vitro* neutralizing assays (21). However, *in vivo* experiments are inherently more cumbersome and expensive and are not useful for the screening of large potential patient populations.

Currently, AAV gene therapy is rapidly expanding with more than 200 ongoing or completer clinical trials (www.clinicaltrials.gov). In my view, going forward, it will be essential that regulatory agencies such as the FDA and the EMA will create standardized *in vitro* assays for each AAV serotype/variant. These assays should be sensitive and rely on laboratory equipment that is commonly available in academic laboratories. Ideally, these assays will be validated *in vivo* in mice and NHPs. Importantly, the assays should include commercially available reagents, such as a neutralizing, monoclonal antibody, to allow the validation of the assay in each laboratory using it. Only standardized assays will allow a valid comparison of NAb levels reported by different groups.

ELISA assays can be used to measure the total levels of antibodies against a specific serotype, whether these antibodies are neutralizing or not. In general, there appears to be a good correlation between total anti-AAV antibody levels and neutralizing antibody (factor) levels (11, 22). However, intravenous IgG (IVIG) appears to also contain non-neutralizing antibodies against AAV that can, in fact, enhance transduction, although the precise mechanism needs to be elucidated (23). As such, the value for using ELISA to determine eligibility for treatment with AAV gene therapy needs to be established fully.

## Approaches to Overcome the Hurdle That Anti-AAV Antibodies Pose to AAV Gene Therapy

In theory, the most appealing approach to overcome the challenge of pre-existing NAbs is to introduce mutations into the AAV capsid that prevent the binding of NAbs. However, the significant cross-reactivity among AAV serotypes (11) shows that this is not an easy feat to achieve. Nonetheless, it has been demonstrated that the introduction of point mutations into the AAV2 capsid can attenuate the sensitivity of these mutant viruses to neutralization (24). However, for most viruses, and presumably for AAV as well, of all the neutralizing antibodies against the virus a large fraction is directed against the receptor binding domain(s). Consequently, mutating residues in the receptor binding region(s) might be best to ameliorate neutralization, but mutation in the receptor binding region(s) are also very likely to affect viral tropism and/or transduction efficiencies.

“Traditional” plasmapheresis, which removes all immunoglobulins, has shown some promise in depleting most NAbs from patient sera, albeit only in patients with low NAb titers (25). Moreover, removing all immunoglobulins has its own drawbacks. More recently Bertin et al. (26) and Orlowski et al. (27) have demonstrated that neutralizing antibodies/factors can be removed *in vitro* by incubating IVIG or human sera with beads that have AAV particles covalently coupled to them. Moreover, my colleagues and I were able to demonstrate that performing hemapheresis with such beads can fully restore liver transduction in animals with NAb titers that without hemapheresis show none to negligible transduction (27). Restoration of transduction of cardiac and especially skeletal muscle was more modest, likely due to rebound of NAbs from the extracellular fluid into the bloodstream. Unfortunately, technical limitations prevented us from performing multiple rounds of hemapheresis over several days (27). Multiple rounds of hemapheresis over the span of several days can easily be performed in humans (25), so it is highly likely that the rebound limitation in rats could be easily overcome in humans. Future tests in large animal models, particularly in non-human primates, could provide proof of principle for the utility of this approach.

Recently, Mingozzi and colleagues took advantage of imlifidase (IdeS), a streptococcal cystein protease, that can cleave IgG into F(ab')_2_ fragments and Fc (28, 29). Treatment of IVIG with IdeS resulted in the complete digestion of total IgG and anti-AAV8 IgG after a 24-h incubation period. In addition, when Leborgne et al. passively immunized mice with human IVIG and 1 day later injected them with AAV8 encoding either secreted Gaussia luciferase or human FIX (hFIX), this completely abrogated Gaussia luciferase or hFIX expression. If, on the other hand, the mice were injected at day 0 first with human IVIG and 30 min. later with IdeS, followed 1 day later by injection of AAV8 encoding secreted Gaussia luciferase or hFIX, the expression levels of the luciferase or hFIX in the blood were indistinguishable from levels in naïve mice (28). Strikingly, not only did IdeS treatment allow transduction of NHPs with pre-existing neutralizing antibodies, it even allowed vector *re-administration* with the same AAV variant (AAV-LK03) (28).

Using IdeZ, a homolog of IdeS produced by a different streptococcal strain, Asokan and colleagues (30) also could demonstrate that administration of IdeZ allows transduction of mice that have been passively immunized with IVIG. Furthermore, they also reported successful transduction of an NHP with pre-existing neutralizing antibodies that had been pre-injected with IdeZ (30).

## Discussion

Pre-existing anti-AAV antibodies are a vexing problem for AAV gene therapy because they can severely limit the patient population that could benefit from AAV gene therapy. This becomes all the more frustrating as the field rapidly advances and many more AAV therapeutics will become available to treat an increasing number of genetic diseases. Nonetheless, over the last couple of years, significant progress has been made in overcoming this formidable obstacle and to extend therapy to patients with pre-existing neutralizing antibodies, either as a result of infection by wild-type AAV or prior treatment with an AAV vector. Because of the ease of use, the IgG cleaving proteases IdeS and IdeZ are particularly attractive to use in AAV gene therapy patients with pre-existing anti-AAV antibodies (28, 30). However, while these proteases cleave all IgGs, they do not remove other transduction inhibitors such as galectin 3-binding protein (18). Plasmapheresis to remove specifically anti-AAV8 antibodies as well as other inhibitory factors (26, 27) is a promising alternative, and only clinical trials will ultimately answer the question, which of the currently available strategies to mitigate the negative effects of anti-AAV antibodies is best for any given therapeutic application. It would, however, be interesting to test with identical human sera whether the addition of IdeS/IdeZ (28, 30) or incubation of the sera with “AAV-beads” (26, 27) has a more positive effect on transduction.

Because of significant differences in assay conditions, a comparison of neutralizing titers obtained by different laboratories is currently impossible. As the use of AAV gene therapy rapidly expands it will, in my view, be absolutely essential that validated assays that are approved by regulatory agencies for any given AAV serotype or variant will be used to determine the amount of neutralizing factors in sera. Until such assays are validated and approved, I urge my colleagues to report very detailed descriptions of their assays used and to report the neutralizing activity of sera/plasma samples as AAV particles neutralized per μl serum/plasma. While imperfect, this should allow at least a “reasonable” comparison of results obtained by different laboratories.

Clearly, a significant amount of future research will be required to “solve” the problem that neutralizing antibodies and factors pose to the expansion of the eligible patient population. Nonetheless, recent developments offer a great deal of optimism that we are on the way to being able to include patients into clinical trials and treatment regimens that owing to the presence of neutralizing antibodies and factors are currently excluded from treatment with AAV gene therapy.

## Author Contributions

The author confirms being the sole contributor of this work and has approved it for publication.

## Conflict of Interest

The author declares that the research was conducted in the absence of any commercial or financial relationships that could be construed as a potential conflict of interest.
